# Women’s experiences following severe perineal trauma: a qualitative study

**DOI:** 10.1186/1472-6874-14-32

**Published:** 2014-02-21

**Authors:** Holly Priddis, Virginia Schmied, Hannah Dahlen

**Affiliations:** 1 School of Nursing and Midwifery, College of Health and Science, University of Western Sydney, Building EB, Parramatta Campus, Locked Bag 1797, Penrith South DC NSW 2751, Australia

## Abstract

**Background:**

Literature reports that the psychological impact for women following severe perineal trauma is extensive and complex, however there is a paucity of research reporting on women’s experience and perspective of how they are cared for during this time. The aim of this study was to explore how women experience and make meaning of living with severe perineal trauma.

**Methods:**

A qualitative interpretive approach using a feminist perspective guided data collection and analysis. Data were collected through semi-structured face to face interviews with twelve women in Sydney, Australia, who had experienced severe perineal trauma during vaginal birth. Thematic analysis was used to analyse the data.

**Results:**

Three main themes were identified: *The Abandoned Mother* describes how women feel vulnerable, exposed and disempowered throughout the labour and birth, suturing, and postpartum period and how these feelings are a direct result of the actions of their health care providers. *The Fractured Fairytale* explores the disconnect between the expectations and reality of the birth experience and immediate postpartum period for women, and how this reality impacts upon their ability to mother their newborn child and the sexual relationship they have with their partner. *A Completely Different Normal* discusses the emotional pathway women travel as they work to rediscover and redefine a new sense of self following severe perineal trauma.

**Conclusion:**

How women are cared for during their labour, birth and postnatal period has a direct impact on how they process, understand and rediscover a new sense of self following severe perineal trauma. Women who experience severe perineal trauma and associated postnatal morbidities undergo a transition as their maternal body boundaries shift, and the trauma to their perineum results in an extended physical opening whereby the internal becomes external, and that creates a continual shift between self and other.

## Background

Severe trauma to the perineum (third and fourth degree tears), the area between the anus and the vagina, can occur spontaneously or as a result of obstetric intervention during vaginal birth [1,2]. Severe perineal trauma (SPT) is defined as a third degree tear, which involves injury to the perineum which extends to the anal sphincter complex; or a fourth degree tear, which involves injury to the perineum involving the external, internal and epithelium of the anal sphincter [3]. It is reported that internationally the incidence of SPT ranges from 05-10% [4,5]. While some women may experience no symptoms, other women may experience any or all symptoms including dyspareunia, stress or urge urinary incontinence, flatus and/or faecal incontinence, and are at risk of developing co-morbidities including pelvic organ prolapse and vesicovaginal fistulas [6-8].

There is a paucity of literature examining the experiences for women who sustain SPT in either the social sciences, midwifery, nursing or medical literature. A meta ethnographic study examining the experiences of women who had sustained a postpartum physical morbidity including SPT identified three major themes: *‘I am broken and a failure’, ‘Dismissed, devalued and disregarded’*, and *‘The practicalities of the unpredictable perineum’*[9]. These themes highlighted that women who experience SPT and associated postpartum morbidities report this has a profound impact on both their physical and psychological wellbeing, and as a result women may experience social isolation and marginalisation particularly those women who experience ongoing morbidity.

Feminist philosophers have explored the physical and psychological transition that occurs during pregnancy and birth, and the impact that this has on how women experience and reshape their sense of self [10-12]. Young describes the physical transition of the maternal body during pregnancy, and how this transformation challenges and shifts previously known body boundaries [12]. It has been described that a shifting of maternal body boundaries occurs during birth, with the pregnant body transitioning to that of the leaking postnatal body, where internal and external boundaries are shifted, and this results in a blurring between self and other [13,14]. Lupton and Schmied have examined women’s loss of self and boundary during the final moments of birth, at which time they become unclear about where they begin and end, and who or what the newborn baby is in relation to the maternal body [13]. When these boundaries do not close again, as in the case of resulting morbidity from SPT, there may be ongoing blurring between the self and other.

The paucity of literature investigating the experiences of women following SPT as a marginalised group, and the impact this has on their role as women, wives and mothers, identified the need for future research to be conducted into this area. The aim of this study was to explore how women experience and make meaning of living with SPT.

## Methods

A qualitative approach was selected as the most appropriate methodology to guide the data collection and analysis for this study. The incorporation of an interpretive feminist perspective allowed for the exploration of gender related oppression and marginalization, and valued the voice of the lived experience of women [15]. Feminist research incorporates a critical research approach that aims to understand and challenge the way in which individuals and groups experience the world, this understanding can facilitate opportunities to bring about change for members of marginalized groups [15,16]. The findings from a meta ethnographic study [9] informed the design of the questions that were asked of the women during the face to face interviews.

Data were collected during face to face interviews with twelve women who had experienced SPT following vaginal birth, data collection continued until saturation was reached. Participants contacted the first author in response to a flyer that was distributed from October 2011 to April 2012 via social media (Facebook) and word of mouth through midwifery colleagues of the first author. In addition, snowball sampling was used as the participants suggested other women who may also be suitable to participate in the study.

The in depth interviews occurred at a time and place convenient to the woman and were recorded using a digital recording device. To ensure informed consent, participation in the interviews was self-determined by the women as a response to distribution of the information sheet. Participants were then asked to read and sign the consent form if they agreed to participate. Participants were advised that all data would be de-identified during transcription of recording to protect identities. The purpose of the interviews was to explore the way women have experienced and understood SPT, and their interactions and experiences with health professionals and health services. Prior to and during the interview process, it was explained to all participants that the first author had sustained severe perineal trauma and as a result experienced long term morbidities. As an “insider” this transparency enabled the ability for the first author to develop rapid rapport and establish relationships with the participants. Each interview was between one to two hours duration. The interviews were semi-structured, using open ended questions (Table 1) [17]. The use of open ended questions enable the complexities of the subjective experience relative to the individual’s own postpartum morbidities and related symptoms, to be uncovered and explored [18,19].

**Table 1:** Interview questions

	
1.	With which baby did you experience a third or fourth degree perineal tear? Can you tell me about your experience?
2.	Following the birth, at what stage were you told that you had sustained a third or fourth degree tear?
3.	Did anyone explain to you how to care for your tear and what treatments you may require in hospital?
4.	Did you have antibiotics?
5.	What advice were you given when you were preparing to discharge the hospital?
6.	Did you see an early childhood nurse/midwife following discharge? Was the third or fourth degree tear spoken about?
7.	What symptoms did you experience that you feel were due to the tear?
8.	How did these symptoms affect your ability to care for your baby? Do household chores? Go out?
9.	How did this experience impact upon your relationship with your partner? How long was it before you felt comfortable to have intercourse? Was it uncomfortable?
10.	What services did you access/ do you access for support or treatment of these symptoms?
11.	Next babies: Have you had any more children following the third or fourth degree tear? Were these babies vaginal births or caesarean? Who made the decision to have a vaginal birth/caesarean? Were you given an episiotomy?
12.	How do you feel about the support you were given in hospital?
13.	Is there anything you would like health workers to know that might help them when they care for women like you?
14.	What do you wish you had known?
15.	How do you see yourself as a person now?

Recordings of interviews were listened to and transcribed verbatim, the transcripts were read thoroughly to allow the researcher to become immersed in the data [20,21]. Data were analysed using thematic analysis [22,23]. Through an iterative process, broad categories were identified as well as variants and exceptions within the data [24]. Through the process of thematic analysis, the first author individually read each interview transcripts to identify patterns of words or statements that related to the aim of the study and these were placed in broad categories. These results were discussed with the co-authors [22,23]. The broad categories were then analysed in detail to identify subthemes which represented the patterns within the broad categories. Overarching themes were then developed to accurately reflect the findings within the data [24].

Ethics approval was obtained by the University of Western Sydney Human Research Ethics Committee. Psuedonyms are used for the participants throughout this paper to protect their identity.

## Results

Twelve women participated in this study, with an average age of 35 years (ranging from 28 to 43 years). Five women were primiparous, seven women had second or subsequent babies. Five of the women were born in Australia, two from the United Kingdom, one from Canada (Table 2).

**Table 2:** Demographics of participants

**Name**	**Age**	**Ethnicity**	**ATSI**	**Number of children**	**Birth no. SPT**	**Degree of trauma**	**Level of education**	**Model of care**	**Time since incidence of SPT**
Ava	32	Australian	No	1	1	3rd	Year 12	Standard	1 year
Sophie	28	Australian	No	1	1	3rd	Year 12	Caseload	18 months
Matilda	32	United Kingdom	No	3	1	4th	University Degree	Standard	7 years
Annabelle	33	Australian	No	3	3	3rd	University Degree	Private midwife	13 months
Chloe	32	Australian	No	2	1	3rd	University Degree	Group midwifery	10 years
Grace	39	Australian	No	1	1	3rd	Tafe	Private midwife	5 months
Samantha	34	Australian	No	2	1	3rd	University Degree	Private obstetric	11 years
Indie	35	Australian	No	1	1	3rd	Tafe	Private midwife	7 weeks
Poppy	33	Australian	No	2	2	3rd	University Degree	Standard	5 years
Lola	39	Canada	No	3	1 +2	3rd x 2 fistula	University Degree	Standard	12 and 10 years
Scarlett	43	United Kingdom	No	1	1	3rd	Tafe	Standard	3 years
Asha	37	Australian	No	3	1 + 3	3rd x 2	University Degree	Private obstetric/Standard	5 years and 13 weeks
Average:	35				1.4				

Eleven women experienced third degree perineal trauma, one woman experienced a fourth degree tear. Two of these women had sustained subsequent third degree tears with one of the women developing a fistula as a result. The length of time that had passed since the participants had sustained SPT ranged from seven week to 12 years. Two of the women gave birth at home, two women had private obstetric care, one woman accessed caseload midwifery care, one woman accessed group midwifery care, and the remaining five women were in the standard public health system where care is generally quite fragmented.

Three main themes were identified: *The Abandoned Mother, The Fractured Fairytale and A Completely Different Normal*.

### The abandoned mother

*The Abandoned Mother* describes how women feel vulnerable, exposed and disempowered throughout the labour and birth, suturing, and postpartum period and how these feelings are a direct result of the actions of their health care providers. This is further explored in the two sub themes below: *Vulnerable and exposed–“I felt like a piece of meat*”, and “*If only they had told me*”.

#### ***Vulnerable and exposed–“I felt like a piece of meat”***

During the interview process, women spoke in depth about their labour, birth and postnatal experiences, reflecting upon the support they received and the professionals that provided care for them during this time. For a small number of women, these people were present and supportive and the women described how they felt cared for, informed, and safe. Ten women however described feeling helpless, out of control and alone, particularly as they reflected upon the moments immediately following birth as the focus of the midwives and obstetricians in the room transitioned from the woman to the new baby.

I was left lying–I just felt like a piece of meat lying on the bed… they gave her to me–it felt like two seconds–and they took her away and wrapped her all up and they spent more time with her and my husband than what they did with me. He (husband) was all wrapped up and cosy with her up in the nice ward upstairs and I was left… (Scarlet)

As women reflected upon the process of perineal suturing and how they were cared for, they described feeling vulnerable, uncomfortable and exposed on both a physical and emotional level. Such feelings of vulnerability and discomfort appeared to be a result of women’s perceptions of the interactions with their care providers. For example, women described how health professionals spoke to and interacted with each other during the labour, birth and suturing process, but rarely communicated directly to the woman herself.

I’m guessing they were midwives, they never actually introduced themselves to me, they just came in and propped my legs up and got the stitch, the needle and thread out, and they had a look, made a couple of attempts and went “the skin just keeps on tearing” and I’ll never forget she, the one woman said to the other, she said “Doctor made this mess, she’ll have to clean it up herself”. And they left me, they didn’t say anything. (Matilda)

I’m kind of insignificant in this whole, you know–I remember these conversations going on around me, I don’t remember anyone physically having a conversation with me. (Samantha)

Women were able to clearly recall the facial expressions, actions and words used by the health professionals as they attended to the repair of the perineal trauma.

And [the doctor] didn’t really want to talk, she just had this disgusted look on her face when she was doing it. It was horrible … she could have said “look it’s not that bad” or something. There was none of that, she just looked like she was someone who was doing her job and not really enjoying it, like “oh (sighed), I can’t believe I have to do this”. (Ava)

As they sought to process and cope during the suturing experience, women responded in various ways, including focussing on the newborn, disassociating from the experience using humour in an attempt to engage the health professional and reconnecting to the process, or being present in the moment which was emotionally confronting.

I don’t remember their faces which is weird. Yeah I don’t remember their faces at all. (Matilda)

I don't know if it was him or the doctor but someone said, man she's just given birth and she’s just cracking jokes like nobody’s business. I think it was my way of coping as well. (Grace)

#### ***“If only they had told me”***

The majority of the women who participated in the study reported that they were often not told about the extent of their perineal trauma. The amount of information and education that women reported receiving regarding the perineal trauma they had sustained, potential symptoms that may develop as a result of perineal morbidity, and access to ongoing treatment varied and contributed to their feelings of being abandoned.

I’m really trying to think of what they did on postnatal ward, I’m trying to think when they actually told me it was a third degree tear. Yeah, I don’t think anyone ever volunteered that information. And if they said a third degree I would have said ‘well what’s that?’ you know. Not knowing about the different levels… (Chloe)

The models of maternity care women accessed appeared to influence the amount of information they received and the support they felt they had and this was most evident where women had continuity of care:

…my midwife had been with me the whole time and yep she explained like when it happened that I had torn really badly. And then it was later that day when I was feeling a bit better that my midwife had come back in to check on me as part of the program there and she went through it all with me again, she was really great, she went over it all with me that day.” (Sophie)

### The fractured fairytale

*The Fractured Fairytale* explores the disconnect between the expectations and reality of the birth experience and immediate postpartum period for women, and how this reality impacts upon their ability to mother their newborn child and the sexual relationship with their partner. The fairytale of motherhood for many women giving birth is challenged by the reality of nipple pain, postpartum bleeding and sleep deprivation. For women who have experienced SPT, the reality they face is a stark contrast to the “fairytale”.

You have this fairy tale where you have your baby and you take it home and everything’s wonderful and then you go round and show it off to everybody. For me it was an effort. It was like I’m too Uncomfortable I can’t be bothered going anywhere. (Scarlet)

There are four subthemes and these will be explored below: *A Broken Body, Achieving a Vaginal Birth, The Contaminated Uncontrolled Body,* and *They Lived Happily Ever After.*

#### ***A broken body***

Following the birth and the suturing experience, women spoke of the ongoing pain they felt in their perineum. Primiparous women often described the level of pain as unexpected, and they were unsure as to whether or not the pain that they were experiencing was a normal level of pain following a vaginal birth. Multiparous women compared the pain, either negatively or positively, to previous experiences.

I couldn’t go to the toilet. I couldn’t sit in the car. And I basically couldn’t do anything–I was standing up and I was so tired, I could barely even lie down, it hurt that much that I just couldn’t think of anything else. (Matilda)

For both primiparous and multiparous women, the pain was placed in context to the amount of impact it had on what the women were and were not able to do, such as moving independently, passing urine and bowel movements, and breastfeeding their newborn. Some women described how they were unable to perform basic parenting tasks in the first few weeks due to the perineal pain and symptoms associated with SPT.

I mean I couldn’t even sit properly on the lounge. I couldn’t get on the floor and do things with him, like I couldn’t sit on the floor and change a nappy. (Poppy)

Women had an expectation that there would be a full recovery and their body would bounce back to normal, that they would be able to function as a “good” mother and wife. When this did not happen they voiced their surprise at the toll birth took on their body and felt more realistic information needed to be given about this.

I think people need to know that birth isn’t this pretty picture. It isn’t the *Home and Away* birth of three pushes and you’re out and you’re up and you’re glamorous the next five seconds. (Asha)

#### ***Achieving a vaginal birth***

Severe perineal trauma is also placed in the context of the birth that they were able to achieve (particularly for women who achieve a vaginal birth after caesarean (VBAC), and the arrival of a well, healthy and happy baby. Vaginal birth appears representative of a woman’s strength and capacity to birth her child, and for some it is a case of “vaginal birth at all costs”. Vaginal birth is for most women part of the fairytale of motherhood and in some ways this compensated them for the feelings of trauma experienced.

And I’m glad I had the experience, I’m glad I had a vaginal birth, I’m glad I didn’t end up in going for a caesarean, I’m glad I birthed him through my own vagina. I can say that even though all of that happened, I did it. And it was horrible in the moment but I can say now that even with all of that I was strong enough. (Matilda)

I guess it was a badge of honour (having a vaginal birth after a caesarean). I had a third degree tear, but I did it. So psychologically that’s how I dealt with it. And I probably, looking back on it now, didn’t give it as much acknowledgement as I should have…’cause yeah, I still tried to do everything as I normally would. (Poppy)

#### ***The contaminated uncontrolled body***

Women described the isolation and embarrassment that came with having what could be described as a contaminated and uncontrolled body. Women often used the word embarrassed to describe how they felt about symptoms that they experienced as a result of SPT, particularly those women experiencing long term urinary and faecal incontinence. Feeling “dirty” and like a “baby with a dirty nappy” resulted in feelings of embarrassment, and in turn impacted upon how they viewed themselves with one woman stating “I just felt hideous”. They felt that there was a stigma associated with incontinence, that “you are dirty and lazy”. The women perceived that it is not culturally appropriate to discuss toileting issues; this lack of discussion resulted in isolation for women as they kept their experiences and symptoms silent. This was the starkest fracturing of the fairytale not only of motherhood but also of womanhood.

Like when you’re a kid if you pooh your pants, there’s this kind of stigma that you’re dirty and lazy. And even when you’re an adult every time it happened I was just like–oh this is filthy, I’m in my twenties and I can’t control myself. I didn’t want to talk to anybody about it, I didn’t even want to talk to the doctor about it. (Matilda)

Some women described feeling shocked at how basic bodily functions were no longer in their control, such as passing their first bowel movement and unexpected episodes of incontinence. This was compounded by the lack of information and education that the majority of women received and the way it collided with their expectation of what would happen following birth.

I remember when I was being sutured I had wind and I was like oh my god. But I think in that first couple of days maybe once didn’t make it to the toilet–urinary. But I didn’t dare tell anyone because how embarrassing… (Poppy)

Further isolation was described by women as they tried to manage their restricted physical capacity, which occurred as a result of perineal pain, and further as they developed strategies to prepare for unexpected episodes of faecal or urinary incontinence.

Like with (my first baby), despite the postnatal depression I was quite happy to go out and do things, all of a sudden I didn’t go ‘cause it was all a bit too hard. ‘Cause what if (my toddler) falls asleep in the car and I can’t pick him up? So yeah maybe I did feel a bit isolated–‘cause I did start to develop postnatal depression when (the baby) was about seven months old… (Poppy)

#### ***They lived happily ever after***

Every fairytale ends with the line, “And they lived happily ever after”. Implicit in this ending is never ending passion, love and a sexually fulfilling life. For women who have experienced SPT the fairy tale is further fractured by changes in the sexual self and the impact this may have on relationships with their partner. Women describe feeling a sense of fear related to the unknown–for women preparing to have intercourse for the first time following SPT, they are fearful of the pain they may experience, fearful of the changes that have happened and how this may impact on their (and their partners) sexual experience. Some women also describe a fear of falling pregnant again and how that will inevitably result in having to give birth again.

It’s almost like trying to do it for the first time…and I’m almost in tears because I’m so scared. He’s so patient but he does go, maybe tonight we can try and I’m like sure. I was so anxious about it–I made myself really sick. I get migraines and I gave myself a massive migraine–the worst and I was vomiting. How classy is that? I’m like all this because I’m thinking I want to have sex. (Grace)

Women who experience pain as a result of SPT, which is mostly described as being due to friction along the scar tissue (two women described experiencing vaginismus), they either delay intercourse as long as possible, or “put up with it”. Some women describe having to take pain relief medication both before and after intercourse to make the experience bearable.

I used to joke–I used to have to have pain relief to have sex, so you know a couple of panadeine, or something stronger if I could find it–nurofen plus was good (laughs). Isn’t it terrible? I mean it’s easier now, I don’t usually take it now before, but after. (Poppy)

For some, having intercourse was described as being an important part of their relationship with their partner, for others they described it as fulfilling a commitment, doing their “duty” as a wife and fulfilling their partners sexual needs. They describe that there is an expectation by themselves and their partner that after 6 weeks, they will be “back to normal”.

Six weeks without sex that’s the little magic number you hear, but to still be eighteen months down the track and it’s very rare that we can achieve intercourse… so it certainly has impacted on our relationship because you know he thought things would be back to normal by now. (Sophie)

For women who were experiencing long term symptomatology such as urinary and faecal incontinence, they described feeling a level of anxiety at their unpredictable bodies and how this impacted upon their sexual identity and intimate relationships with their partners. Intercourse was no longer spontaneous due to fear of an unexpected episode of incontinence, and therefore became scheduled around toileting times to ensure cleanliness.

I do remember having sex a few times and going, I really need to go and being actually really worried about it, and wanting to finish asap and then just I have to go the toilet quickly. Going in and going oh phew, thank God nothing happened, nothing came out. (Lola)

The amount of information regarding perineal pain and symptoms that women shared with their partner varied widely. This appeared to be based upon the length of time that they had been with their partner, the severity of their symptoms (the more symptomatic the less they are likely to share with their partner), and if they have required any ongoing treatment. Women spoke of feeling a sense of concern that their partners would worry about them unnecessarily. The women also wanted to protect the illusion of normality (the fairytale) through this selective sharing of information with their partners.

I think he would probably understand that it’s a sensitive thing. I think that if I had to have surgery I think he’d probably be really sad that I’ve hidden it. Not that I can’t tell him I just don’t really want to right now. Because I’m walking around normally and everything seems quite normal, I don’t think I need to rock that boat. (Lola)

The stark contrast between the expectations and reality for women who have sustained SPT impacts upon both their physical and psychological ability to care for their newborn, their other children, and to fulfil their role as women and wives.

### A completely different normal

*A Completely Different Normal* explores how after women have navigated the treatment pathway maze to try and identify a supportive practitioner/s who can provide an effective treatment option they learn to rediscover and redefine a new sense of self and normality following SPT.

Overwhelmingly, women who experienced long term perineal morbidity and symptomatology described being fearful that any treatment would potentially make their symptoms worse. These women described how they had developed coping strategies over time, embraced a “new kind of normal”, and incorporated these strategies into their daily lives.

I mean at this point I can still cope with it. I’m scared that if they go in there and do something that they might mess it up and make it worse. It doesn’t impact on my life 90 per cent of the time. That other 10 per cent it does, but I can make adjustments to my life to compensate for that. (Lola)

For other women, after seeking multiple treatment and therapies, irrespective of the level of effectiveness of the treatments, women described feeling a sense of resignation and just having to deal with it.

I don’t find it a traumatic thing, now. It’s just what happened. Can’t change it, and really okay I’ve complained about it and figured out well that’s not really doing much and here you go try this tablet, here you go try this cream, but is it just a shut up mechanism? Bandaid affect, that’s how I feel about it. You know what? If these doctors that I’ve spoken to, and they’ve got no answer, then there’s obviously no answer. So deal with it. (Poppy)

#### ***Defining a new sense of self***

For women who sustained SPT, particularly the women who experienced long term morbidities, in processing their experience they were seen to move towards defining a new sense of self. Women reflected upon their experience of SPT as they attempted to understand and normalise the experience. This process appeared to be influenced by the length of time that had passed since the woman had given birth, the level of morbidity that the woman experienced, and the care that they had received. For some women, their experience of pregnancy, labour, birth and the postpartum was one intertwined, complex process, with each element of the journey impacting upon the other. For other women however, their recollection of sustaining SPT and their postpartum experience was understood as a separate experience to that of the pregnancy, labour and birth.

Because I’ve kind of compartmentalised that point in my life as my experience of birth. I’m just of the opinion that you just have to draw a line in the sand sometimes and just say I can choose to look at it negatively or I can choose to look at it positively and see what came out of it. (Matilda)

As women came to grips with their different kind of normal, they learnt to adapt their lifestyle. They realised things should have been different but reached a kind of acceptance. This was seen more with women who had sustained SPT several years ago.

Time went on and I just kind of changed a few things, like stopped wearing G-strings and carried wipes and spare undies around. I just adapted my lifestyle to it. (Lola)

Women attempted to justify their experience by talking about the physiology of the birth process. For some they blame themselves using the words “I’m not stretchy” or “I could have done more”, while others blamed the system, stating “I felt let down”.

No I don’t think that my body has let me down. I think it’s just happened. I think it’s probably just bad luck and these things happen. My babies were not that big. It’s a pretty small hole. I’m not surprised. (Lola)

Some women spoke of how they worked to reconcile the birth that they had hoped for with the reality of their birthing and postnatal experience, and this required a period of processing which led to a resigned acceptance.

… because in a heartbeat I’d do it all again, because she’s my world, but yeah I guess it does make me really sort of upset, I felt I did everything right, but then my body didn’t follow through with what should happen. (Sophie)

I don’t think I’m as confident probably in certain areas. Like I’m always aware of it. I never forget about it. It gets me down sometimes. I just think why can’t everything have just gone normally and healed up. Then on the other hand at least I’m not leaking all the time. It sucks. (Lola)

For women who were able to access a supportive group, meeting women with similar experiences facilitated an opportunity to compare, share strategies and experiences, providing women with a way to comprehend and move towards a “new kind of normal”.

## Discussion

The findings from this research have highlighted the complex physical and psychosocial impact, and the difficulties faced by women who experience SPT during vaginal birth. While current literature focuses on the morbidities that women experience as a result of SPT, the women in this study focused not only on the morbidities but also on how they were treated during the birth, the suturing process and the postpartum period. The model of care, levels of compassion and companionship that women did or did not feel during labour and birth impacted on how they reflected upon, and dealt with, their experience of SPT. Their experience with care providers contributed towards and reinforced the way women viewed their postpartum body as disembodied and disconnected. Limitations of this study included the small number of women that were interviewed in New South Wales only, and that these women were all self-selected, which may represent a limited view. The use of word of mouth and snowball sampling to recruit participants may have resulted in recruitment of women with similar experiences, therefore methods of participant recruitment should be considered for future qualitative studies. Another limitation is that as an insider of the group being researched, it was important that the researcher had an awareness of their place within the research and the potential impact this had upon the interaction with study participants and interpretation of data [25,26]. The use of reflexivity ensured objectivity and authenticity throughout the research process. To further ensure objectivity the interview transcripts, broad categories, subthemes and themes were reviewed and discussed with the co authors.

### Disembodied and disconnected

The women in this study vividly recalled the moment of birth, and the time when they became aware of the damage that had occurred to their perineum following the birth of their baby. They were overwhelmed by the birthing experience and the moment that they met the newborn child, but they were yet to understand the implications of the trauma that they had sustained to their perineum. The transition from self to other–where other is an unknown and unfamiliar self–and the alteration, or for some women, the distortion of body boundaries that occurred in pregnancy, reaches a pinnacle during the birth process [12,13]. At the moment of birth a woman experiences a physical and emotional opening of the self to the other, and the loss of the boundary of what is internal and external occurs as the baby and placenta are born, accompanied by blood and other fluids. While Young [12] suggests that the act of childbirth is a “conclusion”, at which time women undergo a transition to a new self, for women who sustain SPT there is a continuation of this physical trauma associated with birth through the perineum, extending and distorting the boundaries of the known body [12,13]. For some women who experience ongoing urinary, flatus and/or faecal leakage, it may be that closure or sealing of the boundary between the internal and external body is never fully achieved as explored in the subtheme *The contaminated/uncontrolled body*. This creates a contradiction for women who have experienced SPT. Their known body boundaries are permanently altered, yet they seek physical and emotional closure. The disconnected and disembodied sense of self presents multiple challenges to the woman after birth, and this disconnection is in part produced and reproduced by the actions and interactions with health professionals during the time of birth and in the immediate postpartum period [12,27].

It has been argued that existing maternity care is influenced by the works of Descartes (1993) and Cartesian dualism as discussed by Goldberg [28], whereby the mind and body are seen as distinct from each other, the body is seen as an object functioning as separate systems or parts [29,30]. In contrast to the Cartesian dualistic perspective, the work of philosophers such as Merleau-Ponty suggests that the mind and body is in fact a “unified whole”, the body is described as being unique to the individual and anchors the person to their being in the world, using the term ‘the lived body’ [31]. While the writings of Merleau-Ponty have been criticised by feminist scholars as ignoring the concept of gendered subjectivity [9,29,32], it can be argued that the traditional Cartesian view maintains an objectification of the function of the perineum by health professionals, therefore dismissing the psychological impact on women as they face pain and ongoing morbidities following SPT. Women in this study struggled to reattain their known lived body through the physical and emotional barriers that they experienced as a result of the trauma they sustained to their perineum. The women further reported that following the birth the focus of the health professional was on the wellbeing of the newborn baby as described in the theme *The Abandoned Mother* and subtheme *If only they had told me*, and as a result women described feeling like “a piece of meat” with this perspective further reinforcing the dualism and loss of self that women are experiencing.

### The leaking uncontrolled body

Based upon the writings of Merleau-Ponty and the concept of the lived body, it has been suggested that any change to this known self, for example through trauma or illness, can present challenges for the individual in understanding their altered body [33,34]. Literature exploring the impact of disability (physical or intellectual) on the perception of self has described how a separation, or dualism, occurs between the mind and body as the functioning and/or appearance of the disabled body part becomes objectified by the individual as it is unfamiliar and unknown [31,35,36]. This phenomenon was seen during the interviews with the women as explored in the sub theme *A broken body*, who used the terms ‘it’ and ‘thing’ when they referred to their perineum. Such changes can be seen to occur following childbirth, as stated by Young: “The perineum is other, as it no longer functions as it should, however it is attached to me and therefore is mine….” [12], p.50.

Drawing on the work of Kristeva, women who sustain SPT and continue to leak urine and/or faeces may experience abjection, whereby bodily leakage and the altered perineum (described as the abject) contrasts with the cleanliness of the contained pre-pregnant body (non abject), therefore women will work to protect themselves from the threat that the abject presents to their known self (abjection) [10,37]. As stated by Kristeva: “It is thus not the lack of cleanliness or health that causes abjection, but what disturbs identity, system, order.” [10], p.4. Based upon these understandings the negative societal constructs around the leakage of bodily fluids can be seen to add to the abjection that women may experience.

Feminist work exploring constructions of contemporary bodies suggests that a well-managed, contained and controlled body is presented as mature and masculine, and is a stark contrast to the altered, leaking postnatal body that appears uncontrolled and immature [29,38]. The language used by the women in this study reflected this link between uncontrolled bodily functions and immaturity, by referring to faecal incontinence as being dirty like a “toddler” or a “baby”. Similar findings have been reported by Peake et. al who distributed a questionnaire to Australian women who were experiencing urinary incontinence. These women associated leaking urine, and their inability to control episodes of urinary incontinence, with the stereotype of the ‘naughty child’ [39]. Danaher et al. drawing on the work of Foucault suggest that individuals are classified based upon cultural rules, discourses and expectations based upon gender and related bodily functions [40]. For women who are experiencing ongoing urinary or faecal incontinence as a result of SPT, there appears to be a cultural consensus that leaking, messy and ‘dirty’ bodies must be naged and hidden [41-43]. Furthermore, Foucault described self-surveillance as a practice that individuals use to control that which may be perceived as dysfunctional by society to maintain a sense of normality, as technologies of self [40]. The women in this study reported how they made adjustments to their daily living activities to control or minimise unexpected episodes of incontinence to accommodate their unpredictable perineum, as explored in the theme *A Complete Different Normal*. Reflecting upon Kristeva’s concept of abjection, research conducted by Thomas-MacLean examining women’s experiences following mastectomy, found that the women were hesitant to show their scarring to their husbands as a way of protecting their partners from abjection [44]. This reflects the actions of the women in this study who were often reluctant to share information with their partners regarding perineal pain or incontinence so as to protect the partner from experiencing distress and concern, and maintain an appearance of normality [44].

### Sexuality and relationships

Literature has described the impact that childbirth and associated outcomes including breastfeeding, exhaustion and SPT, can have on the sexual relationship between a woman and her partner and this was a major concern for the majority of the women who participated in this study [9,45-47]. As examined in the sub theme *They lived happily ever after*, women described feeling anxious and feared potential pain they may experience, and due to the unpredictable nature of their bodies, were fearful of unexpected episodes of incontinence which impacted upon their ability to engage in spontaneous acts of sexual activity. Similar findings have been reported, suggesting that this level of anxiety and fear impacts not only upon the sexual relationships women engage in with their partners, but their sexual identities as women and wives [9,46,48]. A study conducted by Hipp et al. suggests that following childbirth women are conscious of the needs of their male partners, therefore irrespective of the physical and psychological impact of perineal trauma, women initiate the resumption of sexual activities to fulfil these expectations [49]. Despite this it has been reported that for women experiencing perineal pain and dyspareunia following vaginal birth, there may be a delay in the resumption of intercourse [47,50]. In reflecting upon the changes that occur to the pregnant body, Young describes how awareness may be drawn to a part of the body (such as the pregnant abdomen) due to the uncomfortable sensations or restrictive nature of this object [12]. Similarly, this experience may occur for women who have experienced SPT, they are made aware of the object (the perineum) due to unfamiliar sensations, discomfort or pain due to tenderness and scar tissue, and this may also lead to fear. This awareness may result in women focussing on the object, and therefore unable to enjoy the moment during the act of sexual activity.

### The paradox

The women in this study described feeling surprised and upset when the reality of childbirth and the postnatal period did not match their expectations, resulting in a “fractured fairytale”, however despite this they were grateful to have experienced a vaginal birth. Boon suggests that motherhood is shaped upon social and cultural expectations, whereby when maternal morbidity does occur there is an expectation that it will be accepted as part of the birthing process, and that “The glory of motherhood and the wonder of new life compensating, somehow, for permanent physical debilitation.” [51] p. 193.

For the majority of women who participated in this study, particularly the primiparous women and the woman who had achieved a vaginal birth after caesarean (VBAC), they reflected positively upon their experience of labour and vaginal birth, describing the experience as “empowering” and “beautiful” as described in sub theme *Achieving a vaginal birth*. It seemed paradoxical that women gave accounts of their pleasure of giving birth vaginally, the act that had resulted in their physical trauma, and the emotional distress and how they felt they were treated, and that they were somehow able to resolve this. There is evidence to suggest that women identify a strong link between vaginal birth, femininity and womanhood, this link is apparent in literature examining birth after trauma and VBAC [46,52-54]. Studies have shown that women report the benefits of vaginal birth as enabling bonding with their newborn infant and assisting in establishing breastfeeding; this is reported as being of particular importance to women who are planning a vaginal birth after caesarean [9,53].

The disconnect between the expectations and reality of labour, birth and parenthood may have psychological implications for women leading to feelings of guilt, anxiety and a sense of loss, which may lead to the development of postnatal depression [55,56]. Literature exploring mothering describes how despite the physical and psychological challenges faced by women there is an expectation by society, and the women themselves, that maternal tasks will be completed by the new mother, and consequently an inability to complete these tasks may impact upon the emotional wellbeing of new mothers [54,57]. Such feelings are reported particularly by women who experience physical trauma during the birthing experience, or have an unexpected birthing outcome, including instrumental birth, caesarean section and postpartum haemorrhage [54,58]. Rubin’s work on the transition to motherhood describes the impact that body image has upon this time of transition, that the new functional body is representative of the successful transition of the woman to the maternal role [59]. The author describes that for women who experience a postnatal body that is unable to be controlled may experience depression, poor self-esteem and “risk of role failure”, and this resonates with the words of the women in this study [59], p. 240.

## Conclusion

The findings of this study describe and explain the experiences for women who have sustained SPT during vaginal birth. The three main themes identified that how women are cared for during their labour, birth and postnatal period has a direct impact on how they understand and rediscover a new sense of self following SPT. The findings indicate that further research is required into the experiences of women with SPT in accessing appropriate health management services in the immediate postnatal period. An evaluation of current services for women who experience ongoing physical morbidities is also important. Recommendations for future practice include the establishment of specialised perineal care clinics that give consistent and collaborative care for women [46,60].

## Competing interests

The authors declare they have no competing interests.

## Authors’ contributions

HP participated in research design, data collection and analysis, design and drafting of manuscript as a component of a doctoral study. VS assisted with review of data and manuscript drafts. HD assisted with data collection, review of the data and manuscript drafts. All authors read and approved the final manuscript.

## Pre-publication history

The pre-publication history for this paper can be accessed here:

http://www.biomedcentral.com/1472-6874/14/32/prepub

## References

[B1] Dahlen H Homer C Cooke M Upton A Nunn R Brodrick B Perineal outcomes and maternal comfort related to the application of perineal warm packs in the second stage of labor. A randomized controlled trial Birth 2007 34 4 282 290 10.1111/j.1523-536X.2007.00186.x 18021143

[B2] Fernando RJ Sultan AHH Kettle C Thakar R Radley S Methods of repair for obstetric anal sphincter injury Cochrane Database Syst Rev 2006 3 *Art No: CD002866 DOI:* 10.1002/14651858CD002866pub2 2010 10.1002/14651858.CD002866.pub2 16855993

[B3] RCOG The Management of third-and fourth-degree perineal tears: green-top guideline Book The Management of Third-and Fourth-Degree Perineal Tears 2007 29 City: Green-Top Guideline

[B4] Kettle C Tohill S Perineal care Clin Evid 2008 09 1 17 PMC2907946 19445799

[B5] Baghestan E Irgens L Bordahl P Rasmussen S Risk of recurrence and subsequent deliveries after obstetric anal sphincter injuries BJOG 2011 119 62 69 21985470 10.1111/j.1471-0528.2011.03150.x

[B6] Pauls RN Silva WA Rooney CM Siddighi S Kleeman SD Dryfhout V Karram MM Sexual function following anal sphincteroplasty for fecal incontinence Am J Obstet Gynecol 2007 197 6 618.e1-6 18060952 10.1016/j.ajog.2007.08.011

[B7] Bagade P MacKenzie S Outcomes from medium term follow-up of patients with third and fourth degree perineal tears J Obstet Gynaecol 2010 30 609 612 10.3109/01443615.2010.494205 20701512

[B8] Rathfisch G Dikencik BK Beji NK Comert N Tekirdag AI Kadioglu A Effects of perineal trauma on postpartum sexual function J Adv Nurs 2010 66 2640 2010 10.1111/j.1365-2648.2010.05428.x 20735499

[B9] Priddis H Dahlen H Schmied V Women’s experiences following severe perineal trauma: a metaethnographic synthesis J Adv Nurs 2012 69 4 748 759 23057716 10.1111/jan.12005

[B10] Kristeva J Powers of Horror: An Essay on Abjection 1982 New York: Columbia University Press

[B11] Grosz E Volatile Bodies: Toward a Corporeal Feminism 1994 Bloomington: Indiana University Press

[B12] Young IM Pregnant embodiment: subjectivity and alienation J Med Philo 1984 9 45 62 10.1093/jmp/9.1.45 6726101

[B13] Lupton D Schmied V Splitting bodies/selves: women’s concepts of embodiment at the moment of birth Sociol Health Illness 2013 35 828 841 10.1111/j.1467-9566.2012.01532.x 23094983

[B14] Neiterman E Doing pregnancy: pregnant embodiment as a performance Women’s Stud Int Forum 2012 35 372 383 10.1016/j.wsif.2012.07.004

[B15] Ackerly B True J Doing Feminist Research in Political and Social Science 2010 New York: Palgrave Macmillan

[B16] Shapiro M Setterlund D Cragg C Capturing the complexity of women’s experiences: a mixed-method approach to studying incontinence in older women J Women Social Work 2003 18 21 33 10.1177/0886109902239094

[B17] Hammersley M Atkinson P Ethnography: principles in practice 2007 New York: Routledge

[B18] Clair JM Wasserman J Qualitative methods Book Qualitative Methods 2007 City: Blackwell Reference Online 8

[B19] Curry LA Nembhard IM Bradley EH Qualitative and mixed methods provide unique contributions to outcomes research Circulation 2009 119 1442 1452 10.1161/CIRCULATIONAHA.107.742775 19289649

[B20] Sandelowski M Telling stories: narrative approaches in qualitative research Adv Nurs Sci 1991 8 27 37 10.1111/j.1547-5069.1991.tb00662.x 1916857

[B21] Jackson D Daly J Chang E Schneider Z, Elliott D, LoBiondo-Wood G, Haber J Approaches in qualitative research Nursing Research: Methods, Critical Appraisal and Utilisation 2003 2 Sydney: Mosby 139 153

[B22] Braun V Clarke V Using thematic analysis in psychology Qual Res Psychol 2006 3 77 10.1191/1478088706qp063oa

[B23] Liamputtong P Qualitative data analysis: conceptual and practical considerations Health Promot J Austr 2009 20 133 139 19642962 10.1071/he09133

[B24] Polit DF Hungler BP Essentials of Nursing Research: Methods, appraisal and utilization 1997 4 Philadelphia: Lippincott

[B25] Dwyer SC Buckle JL The space between: On being an insider-outsider in qualitative research 2009

[B26] Kanuha VK “Being” native versus “going native”: conducting social work research as an insider Soc Work 2000 45 439 447 10.1093/sw/45.5.439 11029899

[B27] Salmon D A feminist analysis of women’s experiences of perineal trauma in the immediate post-delivery period Midwifery 1999 15 247 256 10.1054/midw.1999.0182 11216258

[B28] Goldberg L Rethinking the birthing body: Cartesian dualism and perinatal nursing J Adv Nurs 2002 37 446 451 10.1046/j.1365-2648.2002.02111.x 11843983

[B29] Davis-Floyd RE The technocratic, humanistic, and holistic paradigms of childbirth Int J Gynecol Obstet 2001 75 S5 S23 10.1016/S0020-7292(01)00510-0 29645265

[B30] Leder D Medicine and paradigms of embodiment J Med Philo 1984 9 29 43 10.1093/jmp/9.1.29 6726100

[B31] Merleau-Ponty M The phenomenology of perception (translated by Colin Smith) 1962 London: Routledge & Kegan Paul

[B32] Akrich M Pasveer B Embodiment and disembodiment in childbirth narratives Body Soc 2004 10 63 84 10.1177/1357034X04042935

[B33] Schmied V Blurring the boundaries: Breastfeeding as discursive construction and embodied experience 1999 Sydney: University of Technology

[B34] Boughton M Lawler J Embodied self, human biology and experience The body in nursing 1997 Melbourne: Churchill Livingstone

[B35] Edwards S The body as object versus the body as subject: the case of disability Med Health Care Philos 1998 1 47 56 10.1023/A:1009985821964 11081282

[B36] Cadwallader JR Stirring up the sediment: the corporealpedagogies of disabilities Discourse: Stud Cult Polit Educ 2010 31 513 526 10.1080/01596306.2010.504366

[B37] McCabe J Rudge T, Holmes D Subjectivity and embodiment: acknowledging abjection in nursing Abjectly Boundless: Boundaries, Bodies and Health Work 2010 England: Ashgate 213 226

[B38] Braidotti R Conboy K, Medina N, Stanbury S Mothers, Monsters, and Machines Writing on the body: Female embodiment and feminist theory 1997 New York: Columbia University Press

[B39] Peake S Manderson L Potts H “Part and Parcel of Being a Woman”: female urinary incontinence and constructions of control Med Anthropol Q 1999 13 267 285 10.1525/maq.1999.13.3.267 10509310

[B40] Danaher G Schirator T Webb J Understanding Foucault 2000 Australia: Allen Unwin

[B41] Oakley A Fracture: Adventures of a broken body 2007 Bristol: The Policy Press

[B42] Draper J Blurring, moving and broken boundaries: men’s encounters with the pregnant body Sociol Health Illness 2003 25 743 767 10.1046/j.1467-9566.2003.00368.x 19774746

[B43] Jordan J Kirkham M Containing the ‘leaky’ body: female urinary incontinence and formal health care Exploring the Dirty Side of Women’s Health 2007 London: Routledge: 227 241

[B44] Thomas-MacLean R Rudge T, Holmes D Spoiled identities: women’s experiences after mastectomy Abjectly Boundless 2010 Surrey: Ashgate 103 116

[B45] Hicks T Goodall S Quattrone E Lydon-Rochelle M Postpartum sexual functioning and method of delivery: summary of the evidence J Midwifery Womens Health 2004 49 430 436 10.1016/j.jmwh.2004.04.007 15351333

[B46] Williams A Lavender T Richmond DH Tincello DG Women’s experiences after a third degree obstetric anal sphincter tear: a qualitative study Birth 2005 32 129 136 10.1111/j.0730-7659.2005.00356.x 15918870

[B47] McDonald EA Brown SJ Does method of birth make a difference to when women resume sex after childbirth? BJOG 2013 120 823 830 10.1111/1471-0528.12166 23442053

[B48] Grabowska C Squire C Unhappiness after childbirth The Social Context of Birth 2009 United Kingdom: Radcliffe Publishing 236 250

[B49] Hipp LE Low LK van Anders SM Exploring women’s postpartum sexuality: social, psychological, relational, and birth-related contextual factors J Sexual Med 2012 9 2330 2341 10.1111/j.1743-6109.2012.02804.x 22672428

[B50] Rathfisch G Dikencik BK Beji NK Comert N Tekirdag AI Kadioglu A Effects of perineal trauma on postpartum sexual function J Adv Nurs 2012 66 2640 2649 20735499 10.1111/j.1365-2648.2010.05428.x

[B51] Boon S Autobiography by numbers; or, embodying maternal grief Life Writing 2012 9 191 202 10.1080/14484528.2012.667362

[B52] McGrath P Phillips E Vaughan G Speaking out! qualitative insights on the experience of mothers who wanted a vaginal birth after birth by caesarean section Patient 2010 3 25 32 10.2165/11318810-000000000-00000 22273273

[B53] Fenwick J Gamble J Hauck Y Believing in birth–choosing VBAC: the childbirth expectations of a self-selected cohort of Australian women J Clin Nurs 2007 16 1561 1570 10.1111/j.1365-2702.2006.01747.x 17655545

[B54] Elmir R Schmied V Wilkes L Jackson D Separation, failure and temporary relinquishment: women’s experiences of early mothering in the context of emergency hysterectomy J Clin Nurs 2011 21 1119 1127 22176681 10.1111/j.1365-2702.2011.03913.x

[B55] Beck CT Postpartum depression: a meta-synthesis Qual Health Res 2002 12 453 472 10.1177/104973202129120016 11939248

[B56] Knudson-Martin C Silverstein R Suffering in silence: a qualitative meta-data-analysis of postpartum depression J Marital Fam Ther 2009 35 145 158 10.1111/j.1752-0606.2009.00112.x 19302513

[B57] Vallido T Wilkes L Carter B Jackson D Mothering disrupted by illness: a narrative synthesis of qualitative research J Adv Nurs 2010 66 1435 1445 10.1111/j.1365-2648.2010.05350.x 20497266

[B58] Fenwick S Holloway I Alexander J Achieving normality: the key to status passage to motherhood after a caesarean section Midwifery 2009 25 554 563 10.1016/j.midw.2007.10.002 18191007

[B59] Rubin R Attainment of the maternal role: part 1: processes Nurs Res 1967 16 237 245 10.1097/00006199-196716040-00012 5341290

[B60] Thakar R Sultan AH Sultan AH, Thakar R, Fenner DE Postpartum problems and the role of a perineal clinic Perineal and Anal Sphincter Trauma 2007 London: Springer 65 79

